# Anti‐PrP monoclonal antibody as a novel treatment for neurogenesis in mouse model of Alzheimer's disease

**DOI:** 10.1002/brb3.2365

**Published:** 2021-10-21

**Authors:** Ruolin Li, Ming Ren, Yingxin Yu

**Affiliations:** ^1^ Department of Neurology Affiliated Hospital of Jining Medical University, Jining Medical University 89 Guhuai Road Jining Shandong 272029 China; ^2^ Department of neurology Shanghai blue cross brain hospital 2880 Qixin Road, Minhang District Shanghai 201101 China; ^3^ Department of Neurology Chinese PLA General Hospital 28th Fuxing Road, Haidian district Beijing 100048 China

**Keywords:** 6D11, Alzheimer's disease, Aβ, neurogenesis, prion protein

## Abstract

**Background:**

Alzheimer's disease (AD) is the most common degenerative disease characterized by cognitive impairment, memory decline, and language disorder for which there is no effective treatment. Neurogenesis has been indicated in AD and may play an important role in the pathogenesis of AD. Targeting this pathway is a new idea for the treatment of the disease. A recent study reveals that the cellular prion protein (PrP), a receptor for Aβ oligomers, regulates neurogenesis, and its elevated expression is related to cell differentiation. The aim of the present study was to investigate the neuroprotective effects of 6D11 (PrP monoclonal antibody) via neurogenesis promotion in APP/PS1 transgenic mice and Aβ‐induced cell model of AD.

**Methods:**

In the present study, 9‐month‐old male APP/PS1 mice were injected with 6D11. Then, the Morris water maze was used to examine the spatial learning and memory abilities of the mice in both groups, and immunostained was used to assess the level of Aβ, neurogenesis, and neural stem cells (NSCs) differentiation.

**Results:**

6D11 attenuated cognitive deficits in APP/PS1 transgenic mice, which was accompanied by a decrease of the deposition of Aβ. In addition, 6D11 treatment promoted differentiation of the existing hippocampal cells to neurons.

**Conclusions:**

Our findings confirmed that 6D11 has a therapeutic effect in APP/PS1 transgenic AD mouse model and Aβ‐induced AD cell model, and the effect exerted via increase of neurogenesis and cell differentiation by transduction of Aβ peptide signal.

## 1 INTRODUCTION

Alzheimer's disease (AD) is a degenerative disease of the central nervous system that occurs mostly in the elderly and is mainly characterized by cognitive and memory impairment (Verheijen & Sleegers, 2018). The senile plaque (SP) formed by the accumulation of β‐amyloid protein (Aβ) in the brain, the neurofibrillary tangles (NFTs) formed by intracellular tau protein aggregation, and the loss of neurons are the main pathology of the disease (Jeong, 2017; Peric & Annaert, 2015). Although the pathological features of AD have been extensively described, its etiology remains obscure. At present, commonly used drugs can only improve disease‐related cognitive symptoms, but cannot reverse the progress of the disease.

The soluble Aβ oligomer formed by Aβ precursor protein (APP) which is cleaved by β and γ endonuclease is the promoter of AD pathological cascade (Fukumori et al., 2020). Soluble Aβ oligomer damages the synaptic function of neurons associated with early memory of AD, and blocks long‐term potentiation (LTP) of the hippocampus, thereby injures the spatial memory function (Jiang et al., 2020; Nguyen, 2018). Reports have demonstrated that the Aβ and in particular soluble form of amyloid precursor protein plays a neurodevelopmental role, and increase neural stem cells (NSCs) proliferation and thus neurogenesis (Dar & Glazner, 2020; Scopa et al., 2020).

Neurogenesis is an endogenous complex multistep process that involves proliferation, differentiation, and migration of neural precursor cells and produces adult neurons from NSCs, which occurr during embryonic development and throughout adult life (Boldrini et al., 2018; Kempermann et al., 2018; Sorrells et al., 2018). Neurogenesis is found in the subventricular zone (SVZ), the dentate gyrus (DG) of the hippocampus, and the olfactory bulb (Doetsch et al., 1999). Among this, adult hippocampal neurogenesis is involved in higher cognitive function, most notably memory processes, and certain affective behaviors (Kempermann et al., 2015). It has been reported that neurogenesis drops off in AD (Demars et al., 2010; Moreno‐Jimenez et al., 2019). Previous studies also found that the expression of neurogenesis markers is decreased in SVZ and DG regions of postmortem AD patients and AD mice models (Perry et al., 2012; Ziabreva et al., 2006).

Human cytoplasmic prion protein (PrP) is a glycosylphosphatidylinositol (GPI) anchored membrane‐bound glycoprotein, which is highly conserved in mammals such as in normal human tissues and cells, and most abundantly expressed in the nervous system (Manni et al., 2020). Recent studies reported that PrP is involved in the inhibition of hippocampal synaptic plasticity and LTP of Aβ oligomers as a high‐affinity specific binding site for Aβ oligomers (Larson et al., 2012; Um et al., 2012). Monoclonal anti‐Prion antibody (mAb) 6D11 can block hippocampal‐mediated toxicity of Aβ oligomers (Gunther & Strittmatter, 2010; Lauren et al., 2009), and memory impairment in APP/PS1 mouse models requires PrP expression (Gimbel et al., 2010). In addition, PrP is involved in the regulation of neurogenesis (Peralta et al., 2011; Steele et al., 2006), and its increased expression is related to cell differentiation (Lee & Baskakov, 2010). However, it is unclear whether anti‐prion antibody (6D11) has comparable effects against Aβ‐induced AD through regulate neurogenesis.

To address these issues, in the present study we investigated the therapeutic effects of 6D11 in mouse and cell models of AD and found that 6D11 protects the survival of newborn neurons and cell differentiation by transduction of Aβ peptide signal. Our findings suggest that 6D11 may be an effective agent for AD treatment.

## 2 METHODS

### 2.1 NSCs culture, identification, and differentiation

At 14 days of pregnancy, the pregnant mice were subjected to deep anesthesia, under aseptic conditions, and the embryos were isolated from the uterus and then the brains were removed. The hippocampi were dissected under a microscope, cut into small pieces with scissors, and triturated gently with pipette. The tissue was digested with 0.125% trypsin and the digestion was then stopped with DMEM/F12 containing 10% fetal bovine serum (FBS). After centrifugation at 1000 rpm for 5 min, the supernatant was discarded. Cells were resuspended in DMEM/F12 medium containing 20 ng/ml epidermal growth factor (EGF), 20 ng/ml basic fibroblast growth (bFGF), 2% B27 and 1% penicillin streptomycin double antibody. After counting, cell were inoculated in a 25 cm^2^ culture flask at a density of 5 × 10^5^/ml. The primary neurons were cultured for 7 days, and the liquid was changed once every 2 days. Nestin immunofluorescence staining was performed in some cells to identify the NSCs. The proliferation ability of cultured cells was detected with BrdU labeling in some passage cells. Some cells were induced to differentiate with DMEM/F12 containing 10% FBS for 6 days, change the liquid once every 2 days. After differentiation, glial fibrillary acidic protein (GFAP) and NeuN (neuron‐specific nuclear‐binding protein) were stained with immunocytochemistry.

### 2.2 Effects of Aβ on NSCs proliferation, differentiation, and the protective role of 6D11 on NSCs

NSCs were divided into blank control group, Aβ protein group (10 μM Aβ), and 6D11 treatment group (1:100 6D11 pretreatment). Then, the proliferation (the percent ratio of BrdU‐positive cell) and differentiation ability (NeuN and GFAP‐positive cell) ability of each group were tested.

### 2.3 Animals and treatment

APPswe/PS1dE9 double transgenic mice (TG mice) were originally obtained from Model Animal Research Center of Nanjing University and maintained by cross mating the transgenic mice with C57BL/6J mice wild‐type (WT) mice. The genotyping for transgenic mice were performed by using polymerase chain reaction (PCR) with APP primers. All animals were housed on a 12‐h light/12‐h dark cycle and were allowed free access to food and water. All mice were raised and experimental procedures were performed according to the international guidelines for the ethical use of animals. Animal Care Committee of the Weifang Medical University approved the procedures involving animals. TG mice aged 9 months were either treated with anti‐prion protein monoclonal antibody 6D11 or given phosphate‐buffered saline (PBS). Age‐matched WT mice were given the same dose of 6D11 as controls. Note that 5 μl of either the 6D11 or PBS were administered with intracerebroventricular microinjection.

### 2.4 Behavioral studies

For determining the effect of 6D11 treatment, cognition, spatial memory acquisition, and retention were assessed by using the Morris water maze. In the acquisition (hidden‐platform) test, the hidden platform (6 cm in diameter) was kept constant in the middle of one quadrant throughout training. The training included four trials/day for 5 days, with a 5 min interval between two trials. Each trial lasted 60 s or until the mouse climbed onto the hidden platform, and the escape latency was recorded. In this experiment, five groups of animals including 6D11 treated TG mice, PBS treated TG mice, TG mice, 6D11 treated WT mice, and WT mice were used.

### 2.5 5‐Bromodeoxyuridine labeling and tissue processing

5‐Bromodeoxyuridine (BrdU) (Sigma, St. Louis, USA) (100 mg/kg/day) was intraperitoneally (i.p.) injected to mice (10 months of age) in each group for 3 consecutive days. For labeling the proliferating cells in hippocampus, mice were sacrificed 1 day after the last injection of BrdU. For labeling differentiation of hippocampal proliferating cells, mice were sacrificed 28 days after the last BrdU injection.

Mice were anesthetized with 10% chloral hydrate (4 ml/kg, i.p.) and perfused with 0.01 M PBS (pH 7.4), and the brains were removed. The right hemisphere was immersion‐fixed in 4% paraformaldehyde for at least 24 h, while the left hemisphere was fresh frozen for measurements of PrP. The brains were placed in 20% sucrose in 0.1 M PB at 4°C for 24 h. The brains were then sliced into 40 μm thickness coronal sections with a cryostat.

### 2.6 Immunohistochemistry

The free‐floating sections were first incubated with 5% goat serum and 1% Triton X‐100 in 0.01 M PBS for 1 h, respectively, followed by incubation with primary antibody against Aβ (1:100; Abcam, Cambridge, UK) at 4°C overnight. The sections were then rinsed with 0.01 M PBS for 10 min × 3 and incubated with biotin labeled secondary antibody (KPL, Maryland, USA) at room temperature for 2 h. All sections were rinsed 0.01 M PBS for 10 min × 3 and then incubated with HRP‐conjugated streptavidin (BBI Life Sciences, Shanghai, China) at room temperature for 2 h. The staining was visualized using 3,3′‐diaminobenzidine (DAB), and sections were mounted on slides. The specimens were examined under a Leica microscope.

### 2.7 BrdU immunohistochemistry and quantification

For labeling BrdU, free‐floating sections were first incubated in 2N HCl for 30 min at 37°C to denature DNA and neutralized in 0.1 M borate buffer (pH 8.5) for 10 min. The sections were then incubated with 5% goat serum and 1% Triton X‐100 in 0.01 M PBS for 30 min, respectively, followed by incubation with mouse anti‐BrdU antibody (1:700; Sigma) at 4°C overnight, and then biotin‐labeled anti‐mouse IgG (KPL) and HRP‐conjugated streptavidin (BBI Life Sciences) at room temperature for 2 h, respectively. Between each incubation, the brains sections were rinsed three times in 0.01 M PBS for 10 min, and the staining was visualized with DAB.

### 2.8 Double‐label immunofluorescence and quantification

For determining the relative distribution of new‐proliferating cells after 28 days of survival, brain sections of the right hemisphere were used for double‐staining of BrdU+NeuN (a neuronal marker) as well as BrdU+GFAP (an astroglia marker). Free‐floating sections were incubated at 4°C overnight with either mouse anti‐BrdU (1:700; Sigma) + rabbit anti‐NeuN (1:300; Chemicon, Temecula, CA, USA) or mouse anti‐BrdU ((1:700; Sigma) + rabbit anti‐GFAP (1:1000; Abcam) primary antibodies. After rinsing with PBS, the sections were incubated at room temperature for 2 h with either goat anti mouse IgG‐Alexa Fluro 488 + goat or anti rabbit IgG‐Alexa Fluro 594 secondary antibodies. Finally, all sections were mounted with fluorescent mounting medium.

Fluorescence signals were observed under an Olympus FV5‐PSU confocal microscope. Colocalization of BrdU with either NeuN or GFAP‐positive cells were analyzed. The number of BrdU‐, BrdU+NeuN‐, and BrdU+ GFAP‐positive cells in the hippocampus of each stained section was counted, and the percentage of BrdU‐positive cells colocalize with NeuN or GFAP was calculated.

### 2.9 Western blot

Tissues dissected from hippocampus of mice were homogenized in radioimmunoprecipitation assay (RIPA) buffer containing protease inhibitor. Protein concentrations were determined by using the Bicinchoninic acid assay. Samples were electrophoresed on 10% SDS‐polyacrylamide gels and then transferred to polyvinylidene difluoride (PVDF) membranes. The membranes were blocked with 5% nonfat milk in Tris‐buffered saline Tween 20 (TBS‐T) for 2 h at room temperature, then incubated overnight at 4°C with the following primary antibodies: PrP (1:1000; Abcam), GAPDH (1:500; Boster, Wuhan, China). Then, the membranes were incubated with HRP‐conjugated secondary antibodies (1:1000; Boster) at room temperature for 2 h. The protein bands were visualized with the ECL detection system and quantified with Image J software.

### 2.10 Data analysis

Prism 6 software (GraphPad Inc., La Jolla, CA, USA) was used for statistical analysis. Results were presented as means ± SD from at least three repeats. Statistical analysis was performed by using analysis of variance followed by a Bonferroni's post‐hoc correction for multiple comparisons. Differences were considered significant at *p* < .05.

## 3 RESULTS

### 3.1 Injection improves cognitive impairment in TG mice

**FIGURE 1:**
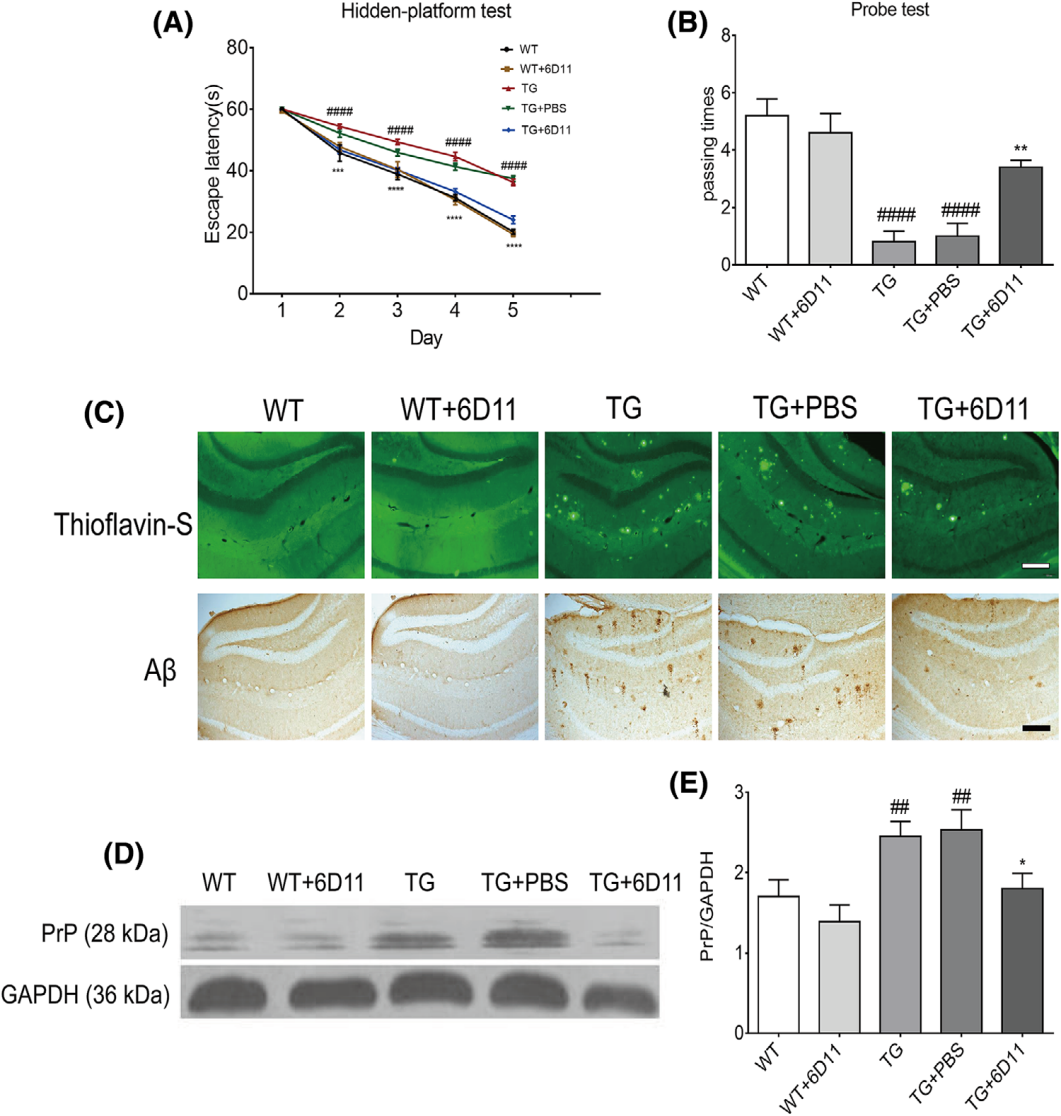
6D11 treatment reverses cognitive deficits and restores the amyloid pathology and prion protein expression in transgenic (TG) mice. Wild‐type (WT) and TG mice (9 months old) were treated with 6D11. (a) Escape latency in the hidden‐platform test increased in TG mice when compared with WT mice, and 6D11 treatment shortened this time. Results are shown as means ± SD. (b) Passing times in the probe test decreased in TG mice when compared with WT mice, and 6D11 treatment prolonged this time. Results are shown as means ± SD. (c) Amyloid pathology in the hippocampus of each group mice. Aβ plaques were stained with thioflavin‐S (ThS) and immunostained with anti‐Aβ antibodies. The number of Aβ plaques in the hippocampus of TG mice was increased when compared with WT mice, and 6D11 treatment reverses the increase. Scale bar = 50 μm. (d) 6D11 treatment suppressed the prion protein (PrP) levels in TG mice. Western blotting analysis of PrP levels in extracts from hippocampus of mice. Level of GAPDH was used as the control. (e) Quantified results of western blotting of PrP levels expressed as means ± SD. ^##^ *p*<.01, ^####^ *p*<.0001 versus WT mice; *p < .05, **p < .01, ****p < .0001 versus TG mice

Morris water maze system was used to detect the learning and memory function of mice. The results showed that the escape latency was significantly prolonged in the TG group compared with that of the WT group. The difference was statistically significant, indicating the defect of learning and memory in TG mice. Compared with TG group, the escape latency of 6D11 treated TG mice was shortened. The difference was statistically significant. The results showed that 6D11 improves the learning and memory function of AD mice (Figure 1a,b).

### 3.2 Treatment decreases the number of amyloid pathology and the expression of prion protein in TG mice

The number of hippocampal Aβ plaques was used to confirm the different stages of AD amyloid pathology. Thioflavin‐S staining and immunohistochemistry of tissue sections revealed that the number of hippocampal Aβ plaques in TG mice was significantly higher than in the WT mice and 6D11 treated TG mice (Figure 1c). The results showed that 6D11 improves the amyloid pathology of AD mice.

Western blot result showed that 6D11 treatment induces a significant decrease in protein levels of prion protein (Figure 1d,e). This indicated the protective effects of 6D11.

### 3.3 6D11 treatment protects the survival of newborn neurons in the hippocampus of TG mice

Cell proliferation in hippocampus of the mice was labeled by BrdU staining. BrdU was used as a DNA precursor analogue incorporated into S phase cells. Immunoreactive products were located in the nucleus and brownish yellow after DAB staining. BrdU‐positive cells in the hippocampus are often tightly arranged and clustered. The cells have irregular shapes, mostly oval or round.

**FIGURE 2:**
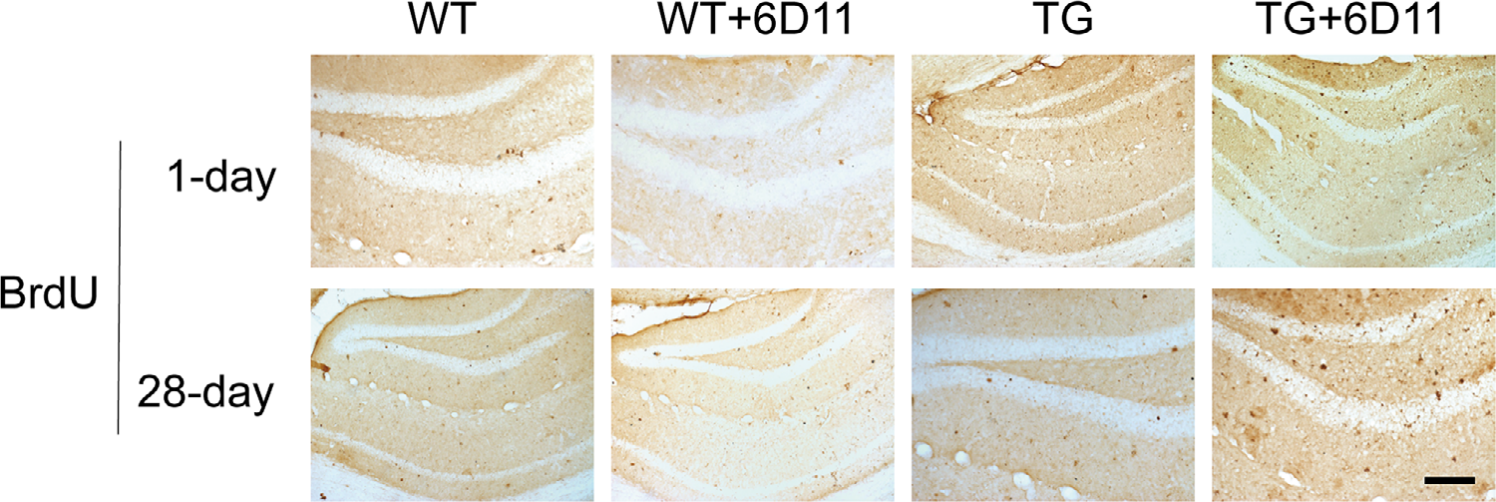
6D11 increases cell proliferation in the hippocampus of transgenic (TG) mice. Cell proliferation labeled by 5‐bromodeoxyuridine (BrdU) staining in the hippocampus of the mice. Mice were sacrificed at day 1 and day 28 after the last BrdU injection for BrdU immunostaining. The number of 1‐day‐old BrdU‐positive cells in hippocampus of TG mice was increased when compared with WT mice. There was no difference between 6D11 treated TG mice and TG mice. The number of 28‐day‐old BrdU‐positive cells in hippocampus of 6D11 treated TG mice was increased when compared with TG mice. Scale bar = 100 μm

The number of 1‐day‐old BrdU‐positive cells in hippocampus of TG mice was increased when compared with WT mice. But there was no difference when compared with 6D11 treated TG mice (Figure 2). The number of 28‐day‐old BrdU‐positive cells in the hippocampus, including dentate gyrus (DG), CA1, and CA3 was significantly higher in the 6D11 treated TG mice than that in the TG mice. These differences were not observed in the WT mice compared with 6D11 treated TG mice (Figure 2). It is suggested that 6D11 intervention does not affect neurogenesis in TG mice, but it can protect the survival of newborn neurons in TG mice.

### 3.4 6D11 treatment increases the number of existing hippocampal cells that differentiate to neurons

**FIGURE 3:**
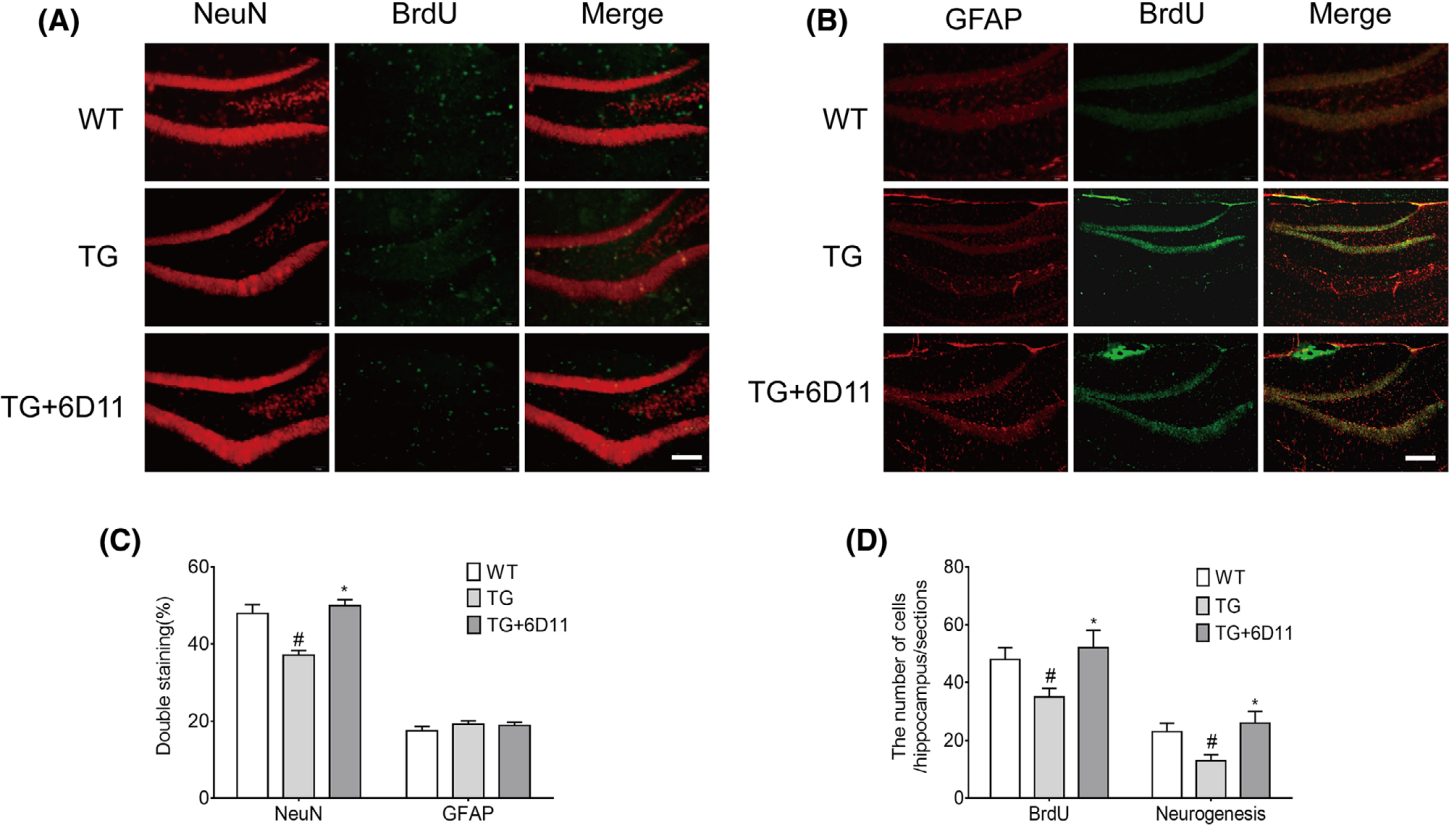
6D11 treatment increases the number of existing hippocampal cells differentiate to neurons. Mice were injected with 5‐bromodeoxyuridine (BrdU) at 9 months old, and sacrificed at day 28 after the last BrdU injection. (a) The number of BrdU‐positive cells in the hippocampus and that differentiate into neurons in 6D11 treated transgenic (TG) mice was significantly increased compared to the TG mice. (b) The number of BrdU‐positive cells in the hippocampus and that differentiate into glia cells in6D11‐treated TG mice was no different when compared to the TG mice. (c) Proportion of BrdU‐positive cells expressing NeuN and GFAP in each mice group. (d) Number of existing hippocampus BrdU‐positive cells and neurogenesis (BrdU+NeuN double‐positive cells) in each mice group. Results were expressed as means ± SD. Scale bar = 100 μm. ^#^ *p <* .05 versus wild‐type (WT) mice; ^*^ *p <* .05 versus TG mice

The phenotype of newborn cells in hippocampus includes neurons (BrdU+NeuN) and glial cells (BrdU+GFAP). The proportion of differentiating BrdU‐positive cells in TG mice was lower when compared with WT mice, and 6D11 treated TG mice increased the proportion of differentiating BrdU‐positive cell. The number of BrdU‐positive cells in the hippocampus and that differentiate into neurons in 6D11 treated TG mice are significantly increased compared to the TG mice (Figure 3), indicating that the pathological changes of AD may hinder the differentiation of neural precursor cells into neurons, then affect brain function, and 6D11 may improve this situation.

### 3.5 Isolation, culture, and identification of NSCs

**FIGURE 4:**
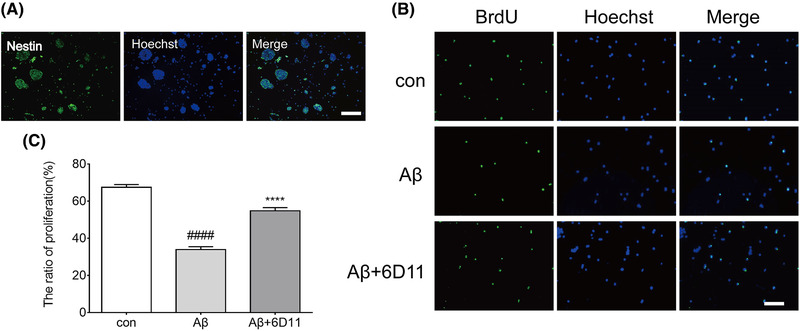
6D11 protected Aβ‐induced neural stem cells proliferation inhibition. (a) Neurosphere was positive for nestin defined by immunofluorescence staining. Scale bar = 100 μm. (b) Expression of 5‐bromodeoxyuridine (BrdU) (green) was detected by immunofluorescence staining. Scale bar = 100 μm. (c) The ratio of proliferation of neural stem cells. The proliferation ratio of Aβ group was decreased when compared with control group. 6D11 pretreatment enhanced proliferation ratio. ^####^ *p <* .05 versus con; ^****^ *p <* .05 versus Aβ group

Both primary and subcultured NSCs can rapidly proliferate, and then form neurospheres. Under the microscope, the neurospheres were observed to be translucent, with regular shapes, neat edges, clear boundaries, tight alignment, and suspension growth in the culture medium. After being identified by immunofluorescence, neurospheres express NSCs specific markers nestin (Figure 4a).

### 3.6 6D11 against Aβ‐induced inhibition of NSCs proliferation and compromised differentiation into neurons

To determine the protective effect of 6D11, we evaluated cell viability with the 3‐(4,5‐dimethylthiazol‐2‐yl)‐2,5‐diphenyltetrazolium bromide (MTT) and lactate dehydrogenase (LDH) assays, respectively. We found that cell viability was reduced and cytotoxicity was enhanced in primary neurons by treating with Aβ (10 μM) for 96 h. 6D11 pretreatment dose was selected 1:10, 1:50, 1:100, 1:200, 1:500, and 1:1000, and detection was conducted at 96 h after Aβ treatment. Our results showed that 6D11 treatment reversed the effect in a dose (1:10, 1:50, 1:100, 1:200)‐dependent manner, although this was not the case for 1:500 and 1:1000 treatment (Figure S1). Based on this result, in subsequent experiments cells were pretreated with 1:100 6D11, then Aβ was added for 96 h.

Proliferation ability was detected by using BrdU incorporation. NSCs cultured for 7 days were induced to differentiate with DMEM containing 10% FBS. After 5 days of differentiation, the Aβ protein and 6D11 were administered to the cells, and after 96 h, the proliferation and differentiation ability of each group was determined with immunofluorescence.

**FIGURE 5:**
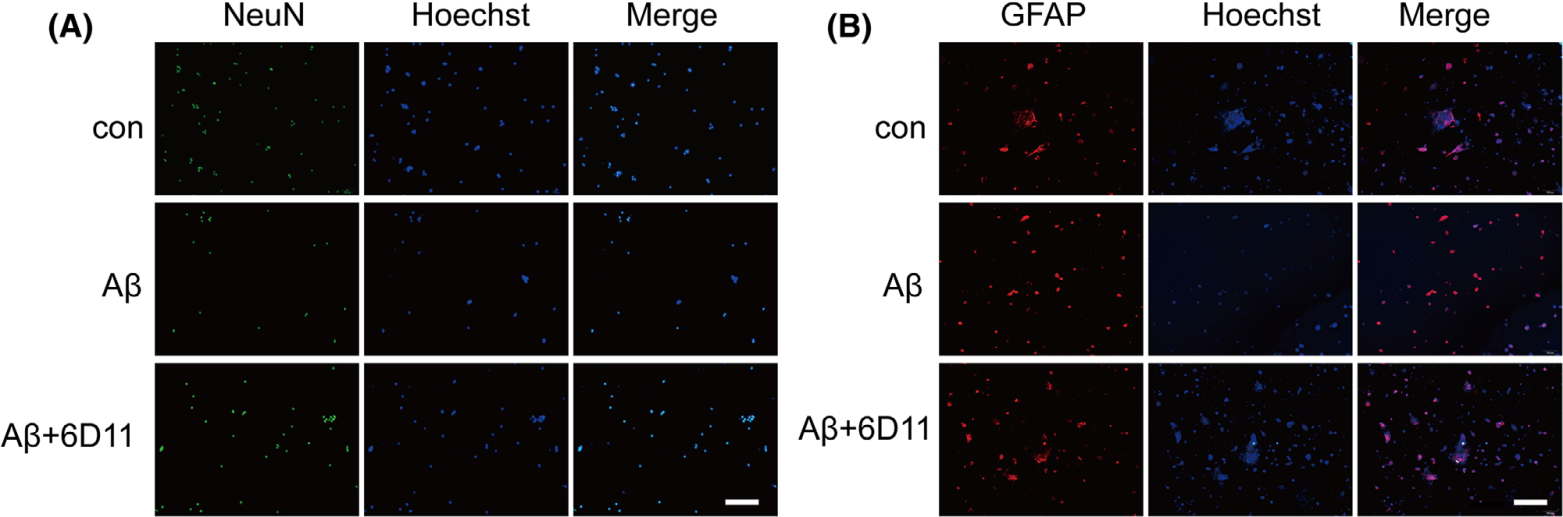
6D11 compromised Aβ‐induced inhibition of neural stem cells (NSCs) differentiation into neurons. Expression of NeuN (a) and glial fibrillary acidic protein (GFAP) (b) were detected by immunofluorescence staining. Scale bar = 100 μm. The proportion of NSCs differentiated into neurons and glial cells was significantly decreased after Aβ added. 6D11 pretreated compromised this reduction

The proliferation ratio of Aβ group was decreased when compared with that of control group, and the difference was statistically significant. 6D11 pretreatment enhanced Aβ‐induced NSCs proliferation inhibition (Figure 4b,c). Otherwise, the ability to differentiate into neurons after added Aβ alone on NSCs was significantly lower than that in the control group. After pre‐treatment of 6D11 for protection, the ability of NSCs to differentiate into neurons was enhanced (Figure 5).

## 4 DISCUSSION

The current study found that 6D11 has a therapeutic effect in APP/PS1 double transgenic AD mouse model and Aβ‐induced AD cell model that is exerted via increased neurogenesis and cell differentiation by transduction of Aβ peptide signal. In this experiment, significant memory impairment and Aβ deposition were found in 9 months old APP/PS1 double transgenic mouse model, which is consistent with the progress of AD disease, and laid the foundation for further experiments.

There are currently few therapeutic options for the treatment of AD. In this study, we investigated 6D11 as a potential agent for AD treatment using cellular and mouse models of AD. Only ∼0.1% of peripherally injected mAb would be expected to cross the blood brain barrier (BBB) and that this small faction can have a significant biological effect. So, intraperitoneal injection requires very large doses of 6D11, and intracerebroventricular microinjection increases the effective utilization of 6D11. 6D11 administration was able to reverse cognitive ability in an AD TG mouse model, as determined by Morris water maze testing, and it can mitigate the deposits of Aβ. In addition, it can also restore APP/PS1 mouse hippocampus cell proliferation, differentiation reduction, and Aβ‐induced neural stem cells proliferation inhibition by decreasing the number of amyloid pathology via inhibition of PrP expression.

It has been found that the PrP is a soluble Aβ oligomer receptor (Lauren et al., 2009), and there is a significant correlation between soluble Aβ and the severity of AD (Pagano et al., 2019; Syvanen et al., 2018). Soluble Aβ oligomers impair neuronal synaptic function which is associated with early memory of AD (Bate & Williams, 2018; Kasza et al., 2017). PrP is a mediator of soluble Aβ causing loss of synaptic function. Transgenic mice with PrP expression showed memory loss, and transgenic mice lacking PrP had no space, learning, and memory loss (Dossena et al., 2008). So, PrP plays a crucial role in the pathogenesis of AD. Chung et al. (2010) applied APP/PS1 transgenic mice intraperitoneally with prion protein antibody 6D11 and demonstrated that prion monoclonal antibody blocks the tangles of soluble Aβ and improves the cognitive function of AD transgenic mice, and our study was consistent with this finding. Kudo et al. (2012) found that the PrP plays a key role in Aβ‐induced neuronal death, suggested that the effect of PrP on AD is not only focused on the synaptic plasticity, but also on neuron death‐another pathological process of AD. The substoichiometric amounts of PrP relative to Aβ will strongly inhibit amyloid fibril formation. The ability of PrP to concentrate Aβ in an oligomeric form and disassemble mature fibers suggests a mechanism by which PrP might confer Aβ toxicity in AD, as oligomers are thought to be the toxic form of Aβ. The ability of PrP that traps Aβ in an oligomeric form is potentially a therapeutic target for the treatment of AD (Nadine et al., 2013).

PrP is particularly expressed at the edge of neural precursor cells that are undergoing differentiation in the central nervous system, and these regions are also the most active regions of neurogenesis (Allison, 2018). Previous studies have shown that the PrP participates in and regulates the neurogenesis of embryonic development, and the differentiated embryonic cells involved in the PrP will develop into neural precursor cells (Peralta et al., 2012, 2015). The high heterogeneity exhibited by neurons and glial cells on PrP expression suggested that PrP plays a key role in the development of neural precursor cells into neurons (Witusik et al., 2007). Moreover, a significant increase in the number of neurons in the mouse overexpressing the PrP indicates that PrP plays a key role in neurogenesis and differentiation (Steele et al., 2006). The present study found that increase neurogenesis in AD mouse model and 6D11 promote the occurrence, maturation, and increase of survival rate of newborn neurons in the brain.

Adult neurogenesis is closely related to brain structural function (Leuner et al., 2006; Park et al., 2002). Studies have showed that the neuronal development of the hippocampal dentate gyrus is closely related to spatial cognitive function. The newborn neurons of the adult hippocampus are thought to replace natural aging or diseased neurons, maximally maintaining the structure and function of the brain (Haughey et al., 2002). Recent studies have found that increased numbers of newborn neurons can improve hippocampal‐dependent learning and memory, while neurological obstruction leads to memory loss. The level of neurogenesis in the hippocampus of AD patients is significantly higher than that in normal people (Jin et al., 2004), which is one of the causes of cognitive decline in AD. The specific molecular mechanisms affecting neurogenesis in AD brain are still unclear, but recent studies have shown that Alzheimer‐type Aβ can significantly reduce the survival of hippocampal newborn neurons (Verret et al., 2007), while anti‐Aβ immunotherapy can significantly promote adult mouse brain neurogenesis.

Our experiment showed that compared with wild‐type mice of the same age, 9‐month‐old APP/PS1 mice showed that the number of BrdU‐positive cells increased in the hippocampus at day 1, and the number of BrdU‐positive cells decreased at day 28. It suggests that the impaired maturation and survival of newborn neurons in AD mice may be the cause of impaired learning and memory. After treatment with 6D11, the number of senile plaques decreased, the phenomenon of neurogenesis increased, and the number of newborn neurons increased significantly, suggesting that stimulating the production of new neurons and increasing their survival to replace the function of aging and dead neurons may be a therapeutic target for AD.

Taken together, our results suggest that 6D11 intervention can significantly improve the pathology and neurobehavioral performance of AD double transgenic mice. The underlying mechanisms of the treatment may include promoting the maturation and survival of newborn neurons in the brain. The in‐depth discussion of its mechanism is for further study.

## CONFLICT OF INTEREST

This work has been funded by National Natural Science Foundation of China (81300923) and Beijing Municipal Science & Technology Commission, PR China (Z16110000516187).

### PEER REVIEW

The peer review history for this article is available at https://publons.com/publon/10.1002/brb3.2365

## Supporting information

Supporting Information

Supporting Information

## Data Availability

Research data are not shared.
