# Supplementary figures and images for: Anti‐PrP monoclonal antibody as a novel treatment for neurogenesis in mouse model of Alzheimer's disease
Source: Brain Behav. 2021 Oct 21;11(11):e2365. doi: 10.1002/brb3.2365 (PMC8613428; doi:10.1002/brb3.2365)

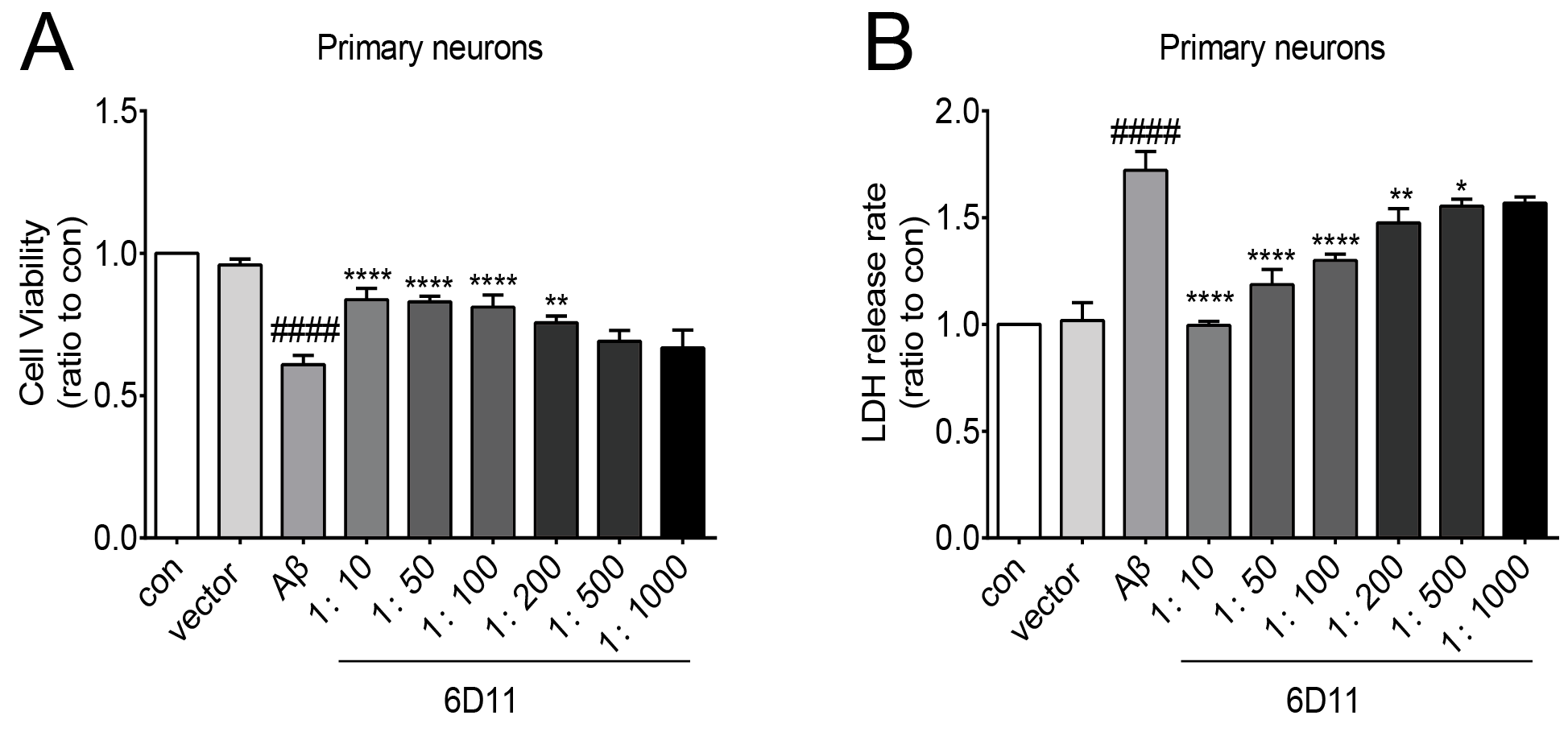

Supplement: Supplementary file 1 — Supporting Information [file BRB3-11-e2365-s001.png]
