# Supplementary material for: Anti‐PrP monoclonal antibody as a novel treatment for neurogenesis in mouse model of Alzheimer's disease
Source: Brain Behav. 2021 Oct 21;11(11):e2365. doi: 10.1002/brb3.2365 (PMC8613428; doi:10.1002/brb3.2365)
Supplement: Supplementary file 2 — Supporting Information [file BRB3-11-e2365-s002.doc]

**Figure S1.** Results were presented as means ± SD from three independent experiments. Six duplication were set for each experiment, and the con group was defined as 1 for normalization process. One-way analysis of variance was adopted to compare the changes of each group. ^####^*P* < 0.0001 *vs.* con; ^*^*P* < 0.05, ^**^*P* < 0.01, ^****^*P* < 0.0001 *vs*. Aβ.
